# The association between rs2228226 and postoperative clinical outcomes in gastric adenocarcinoma: a retrospective study

**DOI:** 10.1186/s12920-025-02102-x

**Published:** 2025-02-11

**Authors:** Haowen Wu, Xinxiong Li, Yuan Dang, Yawei Zhang, Zaizhong Zhang, Bowen Zhang, Qinglong Cai, Lie Wang, Meiping Wang, Chunhong Xiao

**Affiliations:** 1 Department of General Surgery, 900 Hospital of the Joint Logistics Team, Fujian Medical University, 156 Xierhuan Northern Road, Fuzhou, 350025 Fujian China; 2 The First People’s Hospital of Xiaoshan District, Xiaoshan Affiliated Hospital of Wenzhou Medical University, Hangzhou, Zhejiang China; 3 Department of General Surgery, Dongfang Affiliated Hospital of Xiamen University, School of Medicine, Xiamen University, Fuzhou, China; 4 Department of Thyroid Hernia Surgery, Fujian Provincial Hospital, Fuzhou University, Fuzhou, Fujian China; 5 Department of General Surgery, Fuzong Clinical Medical College of Fujian Medical University, Fuzhou, China

**Keywords:** rs2228226, Gastric adenocarcinoma, Prognosis, Biomarkers

## Abstract

**Background:**

This study aims to investigate the differences in postoperative prognosis associated with the single nucleotide polymorphism (SNP) rs2228226 (G > C) in gastric adenocarcinoma (GAC) patients.

**Methods:**

This study enrolled 661 patients with locally advanced (pT4a) GAC after surgery. DNA was extracted from their tissues and genotyped for rs2228226 using a MassARRAY Analyzer. Based on the patients’ clinical and pathological information, a multifactorial Cox regression analysis was performed to assess the correlation between rs2228226 and the clinical prognosis of pT4a GAC patients. Survival differences among patients who received postoperative chemotherapy were also examined according to rs2228226.

**Results:**

After excluding patients with distant metastasis, loss to follow-up, and those not meeting the inclusion criteria, a total of 463 patients with complete data were included. The rs2228226 genotype distribution was as follows: C/C = 57 (12.3%), G/C = 200 (43.2%), and G/G = 206 (44.5%). Patients with the C/C genotype had significantly shorter disease-free survival (DFS = 12 months) and overall survival (OS = 27 months) compared to those with the G/C or G/G genotype (DFS = 19 months, log-rank *P* = 0.003; OS = 35 months, log-rank *P* = 0.002). Further analysis of patients receiving chemotherapy identified the C/C genotype, advanced age, lymph node metastasis, degree of differentiation, and failure to achieve R0 resection as independent risk factors for tumor recurrence and metastasis (*P* < 0.05). The C/C genotype, lymph node metastasis, and tumor recurrence and metastasis were independent risk factors for mortality (*P* < 0.05).

**Conclusions:**

In pT4a GAC patients undergoing postoperative chemotherapy, the C/C genotype at rs2228226 is an independent risk factor for tumor recurrence, metastasis, and death. The rs2228226 (G > C) polymorphism may serve as a potential biomarker for predicting prognosis after chemotherapy in GAC.

## Background

Gastric cancer (GC), a malignant tumor of the digestive system, is the fifth most common cancer globally. Regional variations in incidence and mortality rates of GC are observed, with a higher prevalence in East Asia [1]. The majority of GC cases are gastric adenocarcinoma (GAC), constituting over 90% of all cases. Early-stage GAC often presents with subtle clinical symptoms that can be easily overlooked, resulting in the majority of patients being diagnosed at advanced stages with a high risk of recurrence [2]. At present, the primary treatment approach for gastric cancer is surgery. Nevertheless, the application of targeted drugs for adjuvant chemotherapy remains limited, their efficacy in molecularly typed gastric cancer is suboptimal [3]. These factors pose substantial challenges to both the treatment of gastric cancer and the social security system. Consequently, there is an imperative need for research into the pathogenesis, treatment strategies, and prognostic factors of gastric cancer.

**Fig. 1:**
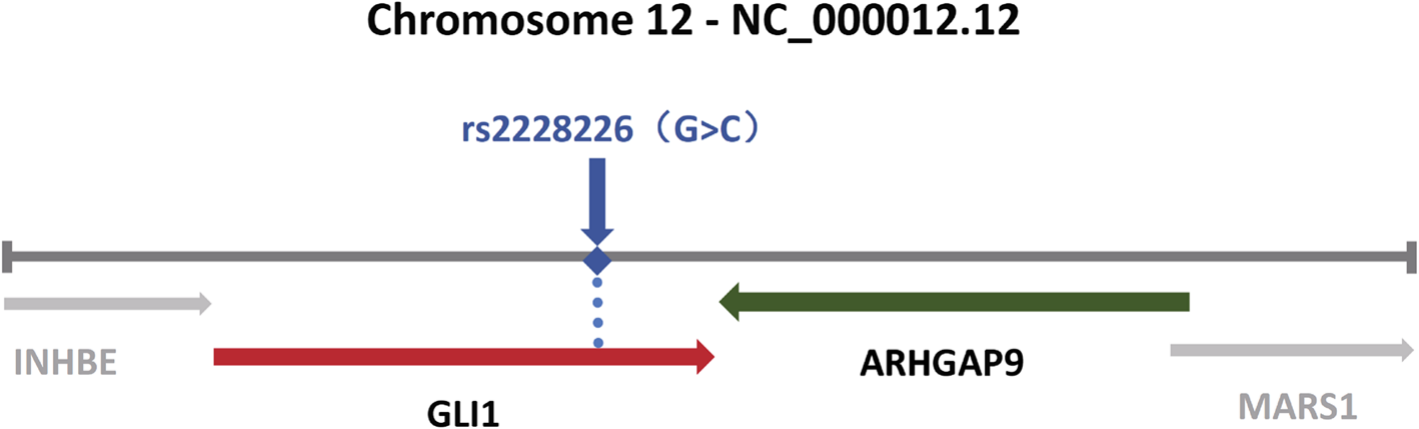
Localization of the rs2228226 SNP on the human chromosome

Single-nucleotide polymorphisms (SNPs) are variations in DNA sequences resulting from mutations at a single nucleotide within the genome. SNPs are closely related to a variety of biological traits that are inextricably linked to the functionality of the gene sequences in which they reside, affecting cancer susceptibility, response to treatment, and survival outcomes [4–7]. The rs2228226 SNP, located within the GLI1 coding region, is situated 500 base pairs downstream of the ARHGAP9 gene (Fig. 1). Furthermore, the ARHGAP9 gene is located in the antisense strand of GLI1 and positioned tail-to-tail on human chromosome 12 [8]. Studies on this SNP have primarily focused on its role within the GLI1 gene. Researchers from Scotland [9] and New Zealand [10] have investigated the association between the rs2228226 SNP and inflammatory bowel disease in Caucasian populations. However, these studies have led to conflicting conclusions regarding risk factor identification. This discrepancy may stem from the underestimation of the rs2228226 polymorphism's potential impact on ARHGAP9 by researchers.

Based on the eighth edition of the tumor-node-metastasis (TNM) staging system for gastric cancer, this study concentrated on locally advanced gastric cancer, specifically the pT4a stage. pT4a GAC specifically targets the serous layer (visceral peritoneum), without extending to adjacent structures or inducing distant metastasis. Tumors invading adjacent structures are often associated with a poor prognosis. Consequently, patients with pT4a GAC who are at a high risk of metastasis are vital participants for inclusion in genetic studies. Through the detection and study of the common SNP rs2228226 in GLI1 and ARHGAP9, we explored its associations with recurrence, metastasis, death, and chemotherapy sensitivity after pT4a GAC, aiming to identify SNPs as potential biomarkers and effective therapeutic targets for prolonging survival in advanced GC patients.

## Methods and materials

### Patients

This study enrolled 661 patients newly diagnosed with GC at the 900th Hospital of the Joint Logistics Support Force in Fujian, China, between July 2003 and December 2009. Prior to surgery, these patients had not received radiotherapy, chemotherapy, or biological therapy and underwent either total gastrectomy or distal gastrectomy. The Department of Pathology at the 900th Hospital of the Joint Logistics Support Force confirmed that all surgical specimens were histopathologically identified as GAC.

GAC tissue samples were collected from surgical patients for genotyping of the rs2228226 SNP (G/G, G/C, C/C), where G represents the major allele and C represents the minor allele. Patient data were extracted from the hospital's information system to construct a comprehensive database encompassing basic patient details, medical history, and personal background. Furthermore, clinical and pathological data were gathered, including tumor site, differentiation and grade, maximum tumor diameter, postoperative TNM classification, lymph node involvement, operative duration, R0 resection status, time to recurrence and metastasis, and survival duration. All patients who received chemotherapy in this study underwent postoperative adjuvant chemotherapy.

According to the 2022 National Comprehensive Cancer Network (NCCN) guidelines for GC, recurrence is defined as the local reemergence of tumors at the original surgical site after radical GC surgery, while metastasis is defined as the spread of cancer to distant sites beyond the primary gastric lesion and regional lymph nodes. Overall survival (OS) was calculated from the surgery date to the date of death or the last follow-up (conducted through November 2014). Disease-free survival (DFS) was measured from the time of surgery to the first occurrence of recurrence, metastasis, or death, or to the last follow-up (through November 2014). The endpoints were determined based on median DFS and median OS. Survival data were primarily collected via telephone interviews and the Social Security Death Index system.

After completing all necessary follow-ups, a total of 104 cases were lost to follow-up, corresponding to a loss rate of 15.7% (104 out of 661). Cases with confirmed distant metastasis (*n* = 79), residual gastric cancer (*n* = 8), non-Han ethnicity (*n* = 2), and severe clinical data deficiencies (*n* = 5) were excluded from the study. In total, the study included 463 patients with complete data. Sample collection was conducted with the approval of the hospital's Medical Ethics Committee.

### DNA extraction and purity assessment

Post-surgical resection, the GC tissue samples were fixed in formalin and embedded in paraffin. Total DNA extraction from the samples was performed using the QIAamp DNA FFPE Tissue Kit (Qiagen GmbH). DNA concentration and purity were assessed using a NanoDrop 2000 spectrophotometer.

### SNP detection

DNA amplification was performed using polymerase chain reaction (PCR). The utilized primer sequences were 5'-CTCCCGAAGGACAGGTATGTAAC-3' (upstream) and 5'-CCCTACTCTTTAGGCACTAGAGTTG-3' (downstream). The reaction mixture contained 1 μl genomic DNA, 0.625 μl of 10 × buffer, 0.325 μl magnesium oxide, 1.0 μl dNTPs, and 0.1 μl thermostable Taq polymerase. The PCR protocol consisted of an initial denaturation at 94 °C for 20 min, followed by 45 cycles of 94 °C for 30 s (denaturation), 56 °C for 30 s (annealing), 72 °C for 1 min (extension), a final extension at 72 °C for 3 min. Subsequently, samples were analyzed using a MassARRAY Analyzer Compact mass spectrometer. The experimental data were analyzed using TYPER software (Sequenom).

### Statistical analysis

Statistical analyses and graphing were conducted using SPSS (Version 22.00) and GraphPad (Version 7.00). Categorical variables were described in terms of frequency counts and percentage composition. Comparisons of categorical data between groups were made using the Mann–Whitney U test or Fisher’s exact test. Adherence of genotype distributions to the Hardy–Weinberg equilibrium was assessed using the chi-squared test. Prognostic influencing factors were evaluated using the Cox proportional hazards regression model for both univariate and multivariate analyses, with calculation of the hazard ratio (HR) and 95% confidence interval (CI). Significance was assessed using a two-tailed test, with the exception of the univariate Cox proportional hazards regression model, which used α = 0.10; all other tests were conducted at α = 0.05.

## Results

### The association between rs2228226 and clinical demographic characteristics

In the postoperative tissue samples from 463 patients with pT4a GAC, the rs2228226 (G > C) genotype distribution was: C/C = 57 (12.3%), G/C = 200 (43.2%), G/G = 206 (44.5%) (Table 1). The study tested for Hardy–Weinberg equilibrium, and *P* > 0.05 indicated that the rs2228226 (G > C) genotype distribution was in genetic equilibrium. The mean age of patients with GAC was 60.43 years, ranging from 28 to 89 years. There was no statistically significant difference in age distribution among the genotypes (*P* > 0.05).

**Table 1:** Data of follow-up patients with pT4a gastric adenocarcinoma and its association with survival

Variables	Patients N(%)	rs2228226genotype(N)(%)			p^*^
		C/C	G/C	G/G	
(Total)	463	57(12.3)	200(43.3)	206(44.5)	
Age					0.700
< 60	212(45.8)	27(12.7)	97(45.8)	92(43.4)	
≥ 60	251(54.2)	34(13.5)	103(41.0)	114(45.4)	
Gender					0.032
Male	341(73.7)	40(11.7)	137(40.2)	164(48.1)	
Female	122(26.3)	17(13.9)	63(51.6)	42(34.4)	
Types of gastrectomy					0.459
Total	281(60.7)	31(11.0)	116(41.3)	134(47.7)	
Distal	146(31.5)	20(13.7)	69(47.3)	57(49.0)	
Proximal	36(7.8)	6(16.7)	15(41.7)	15(41.7)	
Lymph node metastasis					0.582
Yes	357(77.1)	47(13.2)	152(42.6)	158(44.3)	
No	106(22.9)	10(9.4)	48(45.3)	48(45.3)	
Tumor size					0.465
< 5 cm	272(58.7)	35(12.9)	111(40.8)	126(46.3)	
≥ 5 cm	191(41.3)	22(11.5)	89(46.6)	80(41.9)	
Tumor localization					0.035
Upper1/3	160(34.6)	17(10.6)	56(35.0)	87(54.4)	
Middle1/3	74(16.0)	11(14.9)	34(45.9)	29(39.2)	
Lower1/3	220(47.5)	27(12.2)	108(49.1)	85(38.6)	
Mixed	9(1.9)	2(22.2)	2(22.2)	5(55.6)	
Histologic grade					0.151
Poorly	206(44.5)	31(15.0)	92(44.7)	83(40.3)	
Moderately	228(49.2)	25(11.0)	92(40.4)	111(48.7)	
Well	29(6.3)	1(3.4)	16(55.2)	12(41.4)	
Residual tumor					0.033
R0	405(87.5)	49(12.1)	184(45.4)	172(42.5)	
R1	58(12.5)	8(13.8)	16(27.6)	34(58.6)	
Chemotherapy					0.619
Yes	287(62.0)	32(11.1)	125(43.6)	130(43.2)	
No	176(38.0)	25(14.2)	75(42.6)	76(43.2)	
Recurrence or metastases					0.027
Yes	323(69.8)	47(14.6)	129(49.9)	147 (45.5)	
No	140(30.2)	10(7.1)	71 (50.7)	59(42.1)	
Survival					0.056
Alive	144(31.1)	10(6.9)	68(47.2)	66(45.8)	
Dead	319(68.9)	47(14.7)	132(41.4)	140(43.9)	

^*^The *p*-value was calculated using the rank sum test. When the sample size is less than 5, the *p*-value test level *p* < 0.05 is calculated using the Fisher exact probability method

This study included 341 (73.7%) male patients and 122 (26.3%) female patients, with a male-to-female ratio of approximately 2.8:1. A significant difference in sex distribution was observed among the genotypes (*P* < 0.05). Males were more frequently observed to have the G/G genotype, whereas females had a higher prevalence of the C/C and G/C genotypes. No significant differences were found in the distribution of patients across genotypes regarding surgical methods (total gastrectomy, proximal gastrectomy, distal gastrectomy), lymph node metastasis, or tumor size (≥ 5 cm, < 5 cm). However, significant differences in tumor location, achievement of R0 resection, and incidence of recurrent metastasis were noted among patients with different genotypes (*P* < 0.05) (Table 1).

### The association between clinical features and survival status

As of November 2014, 323 out of 463 patients (69.8%) with stage 4a GAC experienced recurrent metastasis as an endpoint event. Furthermore, 319 out of 463 patients (68.9%) experienced death as an endpoint event. The median OS was 33 months, and the median DFS was 21 months. Among patients aged < 60 years, 144 (67.9%) developed recurrent metastasis and 139 (65.6%) died; in contrast, among those aged ≥ 60 years, 179 (71.3%) experienced recurrent metastasis and 180 (71.7%) died. The Cox proportional hazards regression analysis indicated that age was a statistically significant predictor for both OS and DFS (*P* < 0.1) (Table 1).

Among male patients, there were 236 (69.2%) cases of recurrent metastasis and 233 (68.3%) deaths; among female patients, there were 87 (71.3%) cases of recurrent metastasis and 86 (70.5%) deaths. The Cox univariate analysis, with sex as a categorical variable, found no statistically significant association with OS or DFS (*P* > 0.1). The analysis, including other categorical variables, identified lymph node metastasis, tumor differentiation, R0 resection status, chemotherapy, tumor recurrence and metastasis as statistically significant predictors for OS (*P* < 0.1). Consistently, lymph node metastasis, tumor differentiation, R0 resection status, and chemotherapy were statistically significant for DFS (*P* < 0.1).

### Correlation analysis of rs2228226 and survival status

**Fig. 2:**
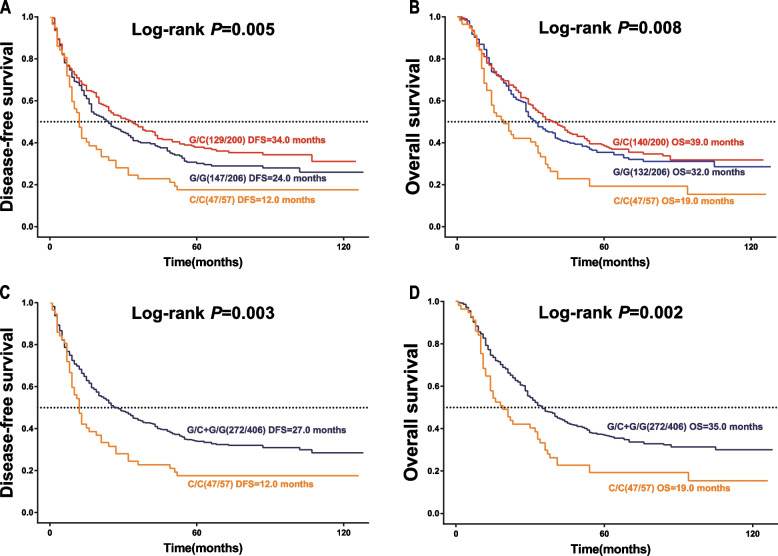
Genetic model analysis of the rs2228226 survival curve. **A**, **B** Additive model Kaplan–Meier curves for DFS and OS based on rs2228226. **C**, **D** Recessive model Kaplan–Meier curves for DFS and OS based on rs2228226. The P-value was determined using the log-rank test

Among the 57 patients with the C/C genotype, 47 (82.5%) experienced recurrent or metastasis, 47 (82.5%) experienced death. Among the 200 patients with the G/C genotype, 129 (64.5%) experienced recurrent metastases, 140 (70.0%) experienced death. Among the 206 patients with the G/G genotype, 147 (71.4%) experienced recurrent metastases, 132 (64.1%) experienced death. rs2228226 established the additive and recessive models and then fitted four Cox regression models (additive OS, recessive OS, additive DFS, and recessive DFS) for testing. The goodness-of-fit test was performed for each of the four models, and their likelihood ratios (-2Log likelihood) were calculated separately. According to the goodness of fit test and clinical experience, the recessive model was finally used for survival analysis. The Kaplan–Meier test indicated that differences in OS and DFS among rs2228226 genotypes were statistically significant (*P* < 0.05) (Fig. 2). The median OS was 19 months for patients with the C/C genotype, 35 months for those with the G/C or G/G genotype, 39 months for those with the G/C genotype, 32 months for those with the G/G genotype. The median DFS was 12 months for patients with the C/C genotype, 27 months for those with the G/C or G/G genotype, 34 months for those with the G/C genotype, 24 months for those with the G/G genotype (Fig. 2).

Among patients experiencing recurrence and metastasis, DFS demonstrated a statistically significant difference between individuals carrying the G allele and those with the C/C genotype (*P* < 0.05). The risk of recurrence and metastasis was decreased by 29.9% for patients with the G allele relative to those with the C/C genotype (adjusted HR = 0.701, 95% CI = 0.511–0.963). Consequently, the C/C genotype was identified as an independent risk factor for tumor recurrence and metastasis. Female sex and advanced age were also identified as risk factors for tumor recurrence and metastasis, the risk ratio of recurrence and metastasis between men and women was about 1:1.2, the risk of recurrence and metastasis increased by 0.7% for each year of age. Lymph node metastasis without R0 resection was an independent risk factor for tumor recurrence and metastasis (*P* < 0.05), resection without R0 was associated with an increase in the risk of recurrence and metastasis by 69.4% (adjusted HR = 1.694, 95%CI = 1.241–2.312). The presence of lymph node metastasis was the greatest risk factor for tumor recurrence and metastasis (adjusted HR = 2.032, 95%CI = 1.500–2.754), and the presence of lymph node metastasis increased the risk of recurrence and metastasis by 103.2%. Poorly differentiated tumors are a risk factor, but patients with moderately differentiated tumors and higher differentiation have a higher risk of recurrence and metastasis. Chemotherapy was a protective factor for tumor recurrence and metastasis (adjusted HR = 0.819, 95%CI = 0.646–1.039), reducing the risk of recurrence and metastasis by 18.1%, but there was no statistical difference (*P* > 0.05) (Table 2).

**Table 2:** Relationship between rs2228226 polymorphisms and survival in patients with pT4a gastric adenocarcinoma surgery

Variables	DFS^a^			OS^b^		
	HR	95%CI	*p*	HR	95%CI	*p*
SNP						
C/C	1.000(reference)		—	1.000(reference)		
G/C + G/G	0.701	0.511–0.963	0.028	0.765	0.558–1.049	0.096
Age	1.007	0.997–1.017	0.146	1.005	0.995–1.015	0.350
Gender						
Male	1.000(reference)			1.000(reference)		
Female	1.216	0.945–1.564	0.128	1.095	0.845–1.419	0.492
Lymph node metastasis						
No	1.000(reference)			1.000(reference)		
Yes	2.032	1.500–2.754	< 0.001	1.554	1.144–2.113	0.005
Histologic grade						
Poorly	1.000(reference)			1.000(reference)		
Moderately	0.777	0.619–0.977	0.031	0.881	0.694–1.118	0.299
Well	0.959	0.611–1.505	0.854	0.848	0.538–1.337	0.479
Residual tumor						
R0	1.000(reference)			1.000(reference)		
R1	1.694	1.241–2.312	0.001	1.332	0.975–1.819	0.072
Chemotherapy						
No	1.000(reference)			1.000(reference)		
Yes	0.819	0.646–1.039	0.100	0.764	0.603–0.968	0.026
Recurrence or metastases						
No	—	—	—	1.000(reference)		
Yes	—	—	—	83.630	34.163–203.721	< 0.001

^a^The relationship between the variable and overall survival (OS) was assessed using Cox univariate analysis. Age was included as a continuous variable in the analysis, and the test level was set at < 0.10

^b^The relationship between the variables and disease-free survival (DFS) was analyzed using Cox univariate analysis. Age was included as a continuous variable in the analysis, and the test level was set at *p* < 0.10

No statistically significant differences in OS were observed between patients carrying the G allele and those with the C/C genotype (*P* > 0.05). Nonetheless, patients with the G allele exhibited a 23.5% reduced risk of death compared to those with the C/C genotype (adjusted HR = 0.765, 95% CI = 0.558–1.049). Female sex, advanced age, and R1 resection were risk factors for death, with a male-to-female mortality risk ratio of approximately 1:1.1, a 0.5% increase in mortality for each year of age. R1 resection increased the risk of death by 33.2%. The presence of lymph node metastasis and tumor recurrence or metastasis were independent risk factors for mortality (*P* < 0.05), lymph node metastasis increased the risk of mortality by 55.4% (adjusted HR = 1.554, 95%CI = 1.144–2.113). Tumor recurrence and metastasis was the greatest risk factor for mortality (adjusted HR = 83.630, 95%CI = 34.163–203.721), tumor recurrence and metastasis directly increased the risk of death by 82 times. Poorly differentiated is a risk factor for tumors, well-differentiated is associated with a lower risk of death than moderately differentiated. Chemotherapy was an independent protective factor for mortality (*P* < 0.05), receiving chemotherapy reduced the risk of mortality by 23.6% (adjusted HR = 0.764, 95% CI = 0.603–0.968) (Table 2).

### The Impact of rs2228226 on the survival of postoperative chemotherapy patients

**Fig. 3:**
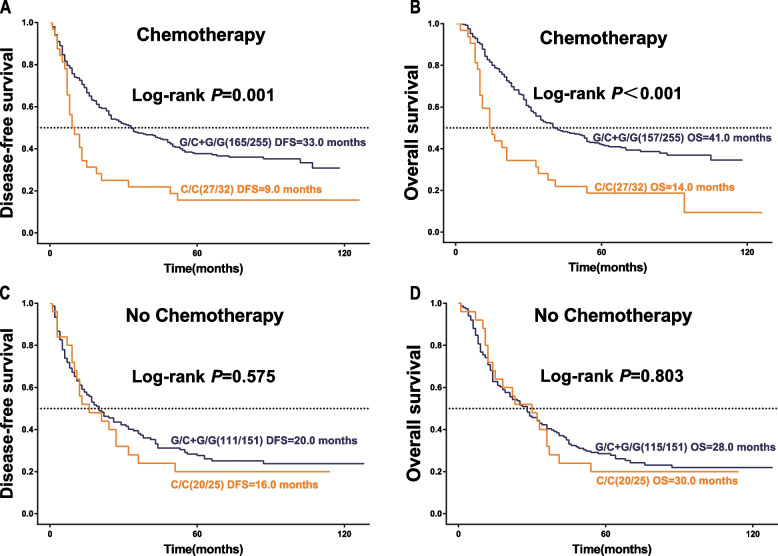
Stratification of survival curves by rs2228226 in relation to postoperative chemotherapy. **A**, **B** Kaplan–Meier curves for DFS and OS in patients receiving surgery and postoperative adjuvant chemotherapy, stratified by rs2228226. **C**, **D** Kaplan–Meier curves for DFS and OS in surgery-only patients, stratified by rs2228226. The log-rank test was used to calculate the *P*-value

Among the 463 patients with stage pT4a GAC, none received preoperative chemotherapy. Postoperatively, 287 (62.0%) of these patients underwent chemotherapy, with an OS of 37 months and DFS of 28 months. A total of 176 (38.0%) patients did not receive postoperative chemotherapy, with an OS of 28 months and DFS of 18.5 months. The postoperative chemotherapy regimen included one or both of the three drugs: cisplatin, epirubicin, and fluorouracil, as well as one or both of the other drugs. The effect of rs2228226 in patients receiving chemotherapy was evaluated by Kaplan-Meyer analysis, which showed a significant difference in OS and DFS (*P* < 0.05) (Fig. 3 AB). However, rs2228226 was not significantly associated with OS and DFS in chemotherapy-naïve patients (*P* > 0.05) (Fig. 3 CD).

In patients experiencing recurrence and metastasis, chemotherapy and carrying the G allele were identified as protective factors for DFS. A statistically significant difference in DFS was observed between patients with and without the G allele (*P* < 0.05). The risk of recurrence and metastasis was reduced by 46.9% (adjusted HR = 0.531, 95% CI = 0.350–0.806). Female sex, advanced age, R1 resection, and lymph node metastasis were the risk factors affecting DFS, the risk ratio of recurrence and metastasis between men and women was about 1:1.2, while the risk of recurrence and metastasis increased by 1.7% for each year of age. The risk of recurrence and metastasis was 69.1% higher in patients with R1 resection than in patients with R0 resection. Lymph node metastasis was the largest risk factor for DFS (adjusted HR = 1.870, 95%CI = 1.258–2.780), which was statistically significant (*P* < 0.05), the risk of recurrence and metastasis was increased by about 87.0% in patients with lymph node metastasis compared with negative patients. Poor differentiation is a risk factor for DFS, but the risk of recurrence and metastasis is higher in patients with a high degree of differentiation (Table 3).

**Table 3:** Analysis of rs2228226 polymorphisms and disease-free survival in patients with gastric adenocarcinoma receiving chemotherapy pT4a

Variables	Univariable		Multivariable^*^	
	HR(95%CI)	*P*	HR(95%CI)	*P*
Genotype				
C/C	1.000		1.000	
G/C + G/G	0.503(0.334,0.758)	0.001	0.531(0.350,0.806)	0.003
Gender				
Male	1.000		1.000	
Female	1.148(0.803,1.588)	0.404	1.196(0.855,1.673)	0.297
Age	1.014(1.001,1.027)	0.038	1.017(1.003,1.030)	0.016
Lymph node metastasis				
No	1.000		1.000	
Yes	2.010(1.366,2.956)	< 0.001	1.870(1.258,2.780)	0.002
Histologic grade				
Poorly	1.000		1.000	
Moderately	0.659(0.490,0.887)	0.006	0.659(0.485,0.896)	0.008
Well	1.113(0.646,1.918)	0.701	1.370(0.782,2.403)	0.271
Residual tumor				
R0	1.000		1.000	
R1	1.641(1.098,2.455)	0.016	1.691(1.118,2.557)	0.013

^*^The *p*-value was calculated using the rank sum test. When the sample size is less than 5, the *p*-value test level *p* < 0.05 is calculated using the Fisher exact probability method

Carrying the G allele was statistically significant for OS compared to the C/C genotype (*P* < 0.05), reducing the risk of death by 42.8% (adjusted HR = 0.572, 95% CI = 0.376–0.870). Male sex, advanced age, R1 resection, and lymph node metastasis were risk factors for OS, the risk ratio of death between men and women was approximately 1.1:1, while the risk of death increased by 1.0% for each year of age. Patients with lymph node metastasis had a 90.3% higher risk of death than those without lymph node metastasis.. Patients with R1 resection had an 11.2% increased risk of death compared with patients with R0 resection. For tumor differentiation, the mortality risk of patients with high and low differentiation decreased by 4.2% and 9.7%, respectively, but the degree of low differentiation was still a risk factor affecting OS. The most significant risk factor for overall survival (OS) was tumor recurrence and metastasis (adjusted HR = 267.121, 95% CI = 37.061–1925.287, *P* < 0.05), which conferred approximately 267 times higher risk of death in patients with tumor recurrence and metastasis compared to those without (Table 4).

**Table 4:** Analysis of rs2228226 polymorphisms and overall survival in patients receiving chemotherapy pT4a gastric adenocarcinoma

Variables	Univariable		Multivariable^*^	
	HR(95%CI)	*P*	HR(95%CI)	*P*
Genotype				
C/C	1.000		1.000	
G/C + G/G	0.458(0.304,0.691)	< 0.001	0.572(0.376,0.870)	0.009
Gender				
Male	1.000		1.000	
Female	1.179(0.848,1.638)	0.327	0.941(0.654,1.354)	0.744
Age	1.017(1.004,1.030)	0.012	1.010(0.997,1.024)	0.123
Lymph node metastasis				
No	1.000		1.000	
Yes	2.126(1.420,3.184)	< 0.001	1.903(1.243,2.915)	0.003
Histologic grade				
Poorly	1.000		1.000	
Moderately	0.638(0.471,0.864)	0.004	0.903(0.649,1.257)	0.544
Well	1.159(0.671,2.000)	0.597	0.958(0.542,1.695)	0.884
Curative effect				
R0	1.000		1.000	
R1	1.738(1.160,2.602)	0.007	1.112(0.730,1.694)	0.622
Recurrence or metastases				
No	1.000		1.000	
Yes	277.769(38.718,1992.779)	< 0.001	267.121(37.061,1925.287)	< 0.001

^*^The *p*-value was calculated using the rank sum test. When the sample size is less than 5, the *p*-value test level *p* < 0.05 is calculated using the Fisher exact probability method

### Survival benefit of rs2228226 in chemotherapy patients

**Fig. 4:**
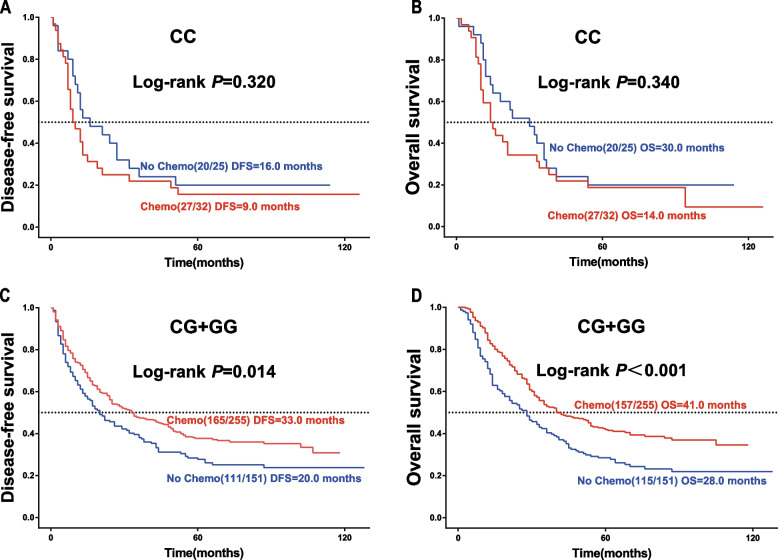
Survival curve stratification of rs2228226 based on chemotherapy. **A**, **B** Kaplan–Meier curves for DFS and OS in patients with the C/C genotype, stratified by postoperative chemotherapy status. **C**, **D** Kaplan–Meier curves for DFS and OS in patients with the G allele, stratified by chemotherapy. The log-rank test was used to calculate the *P*-value

Among the 57 patients with the C/C genotype of pT4a GAC, 27 (84.4%) of the 32 who received chemotherapy experienced tumor recurrence and metastasis (DFS = 9 months), and 27 (84.4%) died (OS = 14 months). Among the 25 patients who underwent surgery alone, 20 (80.0%) experienced tumor recurrence and metastasis (DFS = 16 months), and 20 (80.0%) died (OS = 30 months). Among the 406 patients with G/C or G/G genotype pT4a GAC, 165 (64.7%) of the 255 who received chemotherapy experienced tumor recurrence and metastasis (DFS = 33 months), and 157 (61.6%) died (OS = 41 months). No significant differences in DFS and OS were observed in patients with the C/C genotype who received chemotherapy(*P* > 0.05) (Fig. 4 AB). Among the 406 patients with G/C or G/G genotype pT4a GAC, 165 (64.7%) of the 255 who received chemotherapy experienced tumor recurrence and metastasis (DFS = 33 months), and 157 (61.6%) experienced death (OS = 41 months). Among the 151 patients who underwent radical surgery, 111 (73.5%) experienced tumor recurrence and metastasis (DFS = 20 months), and 115 (76.2%) experienced death (OS = 28 months). The Kaplan–Meier survival analysis demonstrated a statistically significant difference in DFS and OS among patients with G/C or G/G genotype who received chemotherapy (*P* < 0.05) (Fig. 4 CD).

## Discussion

The rs2228226 (G > C) SNP is located on human chromosome 12q13.3. Our analysis determined that patients with pT4a GAC carrying the C/C genotype at the rs2228226 locus exhibited a higher risk of death and recurrent metastasis compared to those with the G/C and G/G genotypes. However, statistical significance was observed solely for recurrent metastasis. These findings suggest that the presence of the G/G or G/C genotype may confer a protective effect against death and recurrent metastasis compared to the C/C genotype.

However, Patients with the G/C and G/G genotypes displayed similar prognoses, exhibiting no significant survival differences. Compared to patients with the G/G genotype, those with the G/C genotype also showed some advantages in DFS and OS. This difference may be related to the genetic characteristics of the rs2228226 locus. The G/C genotype may positively influence tumor recurrence, metastasis, and chemotherapy sensitivity by affecting the function or expression levels of the genes. The G/C genotype may have a potentially protective effect in some ways. Future studies need to further explore the genetic characteristics of rs2228226 and its specific mechanism of action in tumor biology.

In chemotherapy stratification studies, the survival difference between patients with the G allele and those with the C/C genotype may be attributed to the varying susceptibility of rs2228226 to chemotherapy. No significant difference was observed between patients with the C/C genotype who received chemotherapy and those who did not. Consequently, adjuvant chemotherapy does not appear to improve survival outcomes for patients with the C/C genotype. Sikander et al.'s [11] findings regarding the recurrence of rs2228226 (G > C) in stage II and III colon cancer patients within the European population are consistent with our conclusions. Patients with the C/C genotype at rs2228226 were identified as an independent risk factor for recurrence in stage II and III colon cancer patients, with no significant difference in OS. The experimental data corroborate the overall prognostic difference of rs2228226 polymorphism in patients with stage II. and III. colorectal cancer, and also in patients with pT4a gastric adenocarcinoma.

We confirmed that patients with the C/C genotype at rs2228226 were independent risk factors for recurrence, metastasis, and death in chemotherapy-treated patients with pT4a GAC. The risk of recurrence and metastasis was 1.88 times higher, the risk of death was 1.75 times higher, respectively, for patients with the C/C genotype compared to those with the G allele. Advanced age, lymph node metastasis, and inability to achieve R0 resection were all identified as risk factors for recurrent metastasis and death. The inability to achieve R0 resection had a more pronounced impact on the risk of recurrent metastasis, potentially related to postoperative chemotherapy. Tumor recurrence and metastasis emerged as the most significant independent risk factor for death, increasing the risk by 266 times. However, there was some disagreement between this study and Szkandera et al.'s study in the stratification of chemotherapy patients, although it was suggested that the prognostic difference in this SNP was related to chemotherapy, the difference was more pronounced in patients who underwent surgery alone, patients with C/C genotype did not benefit from 5-fluorouracil chemotherapy. Most of the chemotherapy regimens used in the study population were 5-fluorouracil and capecitabine monotherapy, and FOLFOX and XELOX regimens accounted for a very small minority.

The rs2228226 SNP is located within the *GLI1* coding region. Substituting the G nucleotide with C results in the replacement of glutamic acid with glutamine at position 1100 in the *GLI1* protein. This alteration occurs within a conserved region of a critical *GLI1* transactivation domain, thereby altering its charge. Consequently, the transactivation capacity of *GLI1* is reduced [12]. *GLI1* has been positively correlated with cisplatin resistance in GAC [9, 13]. The insensitivity to chemotherapy in patients with the C/C genotype may be attributed to the presence of allele C at this specific SNP site, impacting the relevant *GLI1* functional domain. However, this hypothesis requires validation through additional experiments from various perspectives. Further research is necessary to ascertain whether rs2228226, situated 500 bp downstream of GLI1, modulates the physiological function of ARHGAP9, which is a Rho GTPase-activating protein. ARHGAP9 belongs to a family of proteins critical for the development and treatment of GC [14, 15]. ARHGAP9 contains key functional domains, including Rho GTPase-activating protein (Rho-GAP), Src homology 3 (SH3), PH, and WW domains, which regulate a range of cellular functions [16]. Looking ahead, the use of ARHGAP9 in the GAC is expected to provide a promising avenue for GC-targeted drug research.

This study had several limitations. First, the dataset lacked specific details regarding patients’ chemotherapy regimens. Second, the expression levels of GLI1 and ARHGAP9 in GAC tissues could not be assessed or discussed due to the lack of tissue cDNA for SNP analysis. Future studies should investigate the role of different rs2228226 genotypes in regulating gene expression in GAC cells. Additionally, the retrospective design of the study limits the ability to eliminate selection bias. Our findings suggest that the variable responses to chemotherapy among patients with pT4a GAC may be attributed to the interactive effects of the rs2228226 (G > C) polymorphism in GLI1 and ARHGAP9.

## Conclusion

This study aimed to investigate the clinical prognosis associated with the rs2228226 SNP in patients with GAC following surgery, further assessing the impact of postoperative adjuvant chemotherapy in patients with pT4a GAC. Our results indicated that patients with pT4a GAC harboring the C/C genotype of rs2228226 (G > C) showed no significant benefit from adjuvant chemotherapy. Additionally, the study identified rs2228226 as an independent risk factor for recurrence, metastasis, and death among chemotherapy-treated patients with pT4a GAC. The results suggest that this SNP could serve as a novel genomic-level biomarker for assessing postoperative chemotherapy efficacy in patients with GAC, providing potential implications for the treatment of advanced GAC.

## Data Availability

The data used in the current study are available from the corresponding author on reasonable request.

## Abbreviations

GAC: Gastric adenocarcinoma
GC: Gastric cancer
SNPs: Single-nucleotide polymorphisms
TNM: Tumor-node-metastasis
OS: Overall survival
DFS: Disease-free survival
PCR: Polymerase chain reaction
HR: Hazard ratio
CI: Confidence interval
Rho-GAP: Rho GTPase-activating protein
SH3: Src homology 3
